# Predictors of early response to GnRH and gonadotropin therapy in pediatric patients with suspected dual congenital hypogonadotropic hypogonadism: a retrospective single-center study

**DOI:** 10.3389/fendo.2026.1844974

**Published:** 2026-05-29

**Authors:** Qin Zhang, Yi Wang, Bingyan Cao, Xinmeng Wang, Zheng Yuan, Xinyu Dou, Chunxiu Gong

**Affiliations:** 1 Department of Endocrinology, Genetics and Metabolism, Beijing Children’s Hospital, Capital Medical University, National Center for Children’s Health, Beijing, China; 2 Department of Endocrinology, Capital Center for Children’s Health, Capital Medical University, Beijing, China

**Keywords:** biomarker, congenital hypogonadotropic hypogonadism, GnRH and gonadotropin therapy, pediatric patients, suspected dual defect

## Abstract

**Objective:**

Some patients with congenital hypogonadotropic hypogonadism (CHH) and suspected partial testicular impairment may show variable clinical responses to GnRH and gonadotropin therapy, but predictors of early treatment responsiveness remain unclear. This study aimed to investigate the predictive indicators of early clinical response to GnRH and gonadotropin therapy in pediatric patients with suspected dual CHH.

**Methods:**

This retrospective, single-center study included a total of 37 male suspected dual CHH patients who received GnRH or gonadotropin therapy between January 1, 2010, and January 1, 2025. Based on their early treatment response, patients were divided into two groups: favorable early response (n=20) and suboptimal early response (n=17). Patients who failed to achieve a serum testosterone concentration of ≥200 ng/dL after ≥6 months therapy, had no increase in testicular volume or had no nocturnal emissions during longer follow-up were classified as exhibiting a suboptimal early response. Otherwise, they were classified as exhibiting a favorable early response.

**Results:**

Logistic regression analysis indicated that the level of baseline AMH was positively correlated with a favorable early response (odds ratio = 1.977; 95% confidence interval: 1.010–3.870; p = 0.047). The area under the curve of AMH and testosterone after the hCG prolongation stimulation test was higher, with better sensitivity, specificity, and positive likelihood ratio. When the cut-off value of AMH was 8.9 ng/ml, the sensitivity and specificity were 88.89% and 93.75%, respectively. When the cut-off value of testosterone after the hCG prolongation stimulation test was 51.95 ng/dl, the sensitivity and specificity were 87.5% and 88.89%, respectively.

**Conclusion:**

In this exploratory pediatric cohort with suspected dual CHH, baseline AMH and testosterone after the hCG prolongation test were associated with early response to GnRH and gonadotropin therapy. These findings may help stratify treatment responsiveness, but require validation in larger cohorts with standardized treatment protocols and objective semen-based endpoints.

## 1 Introduction

Congenital hypogonadotropic hypogonadism (CHH) is a disorder caused by impaired synthesis, secretion, or function of gonadotropin-releasing hormone (GnRH) (1), resulting in compromised gonadal activity and hypogonadism. Patients with CHH manifest with micropenis and/or cryptorchidism, absent or delayed puberty, or infertility (2). According to the findings of Sykiotis et al., based on different responses to long-term GnRH replacement therapy, CHH can be divided into three types: triple defect, dual defect (dual CHH), and single hypothalamic defect (3). The dual defect subtype is defined as the absence of sperm production after 21 months of treatment despite achieving normal serum testosterone (T), luteinizing hormone (LH), and follicle-stimulating hormone (FSH) levels. Alternatively, it may be characterized by successful spermatogenesis alongside elevated LH (>17 mIU/mL) and FSH (>14 mIU/mL) levels, despite normal T levels (3). Another study exploring the efficacy of GnRH treatment in patients with CHH found that 10 (4%) prepubertal males showed pulsatile LH release after GnRH pump treatment, but there were no clinical signs of pubertal development (4). Both studies suggested that some patients with congenital hypogonadotropic hypogonadism may also have testicular insufficiency.

The pathogenic mechanism underlying dual defect CHH remains incompletely elucidated. Some researchers consider that it may be related to the defects of genes such as FGFR1, KISS1, and PROKR2, which are expressed simultaneously in the hypothalamus and testes (3). However, not all children with dual HH have the above-mentioned gene variations, so there may be other possible mechanisms. During the physiological male mini-puberty (occurring approximately 1–6 months postnatally), serum gonadotropin (LH and FSH) and testosterone concentrations transiently rise to levels comparable to those observed during mid-puberty. Subsequently, LH and testosterone decline precipitously to undetectable levels, persisting until the reactivation of the hypothalamic–pituitary–gonadal (HPG) axis at puberty onset. In contrast, FSH remains detectable at low but biologically relevant concentrations during this quiescent phase; this residual FSH activity supports continued proliferation of immature Sertoli cells, resulting in age-appropriate, albeit modest, testicular growth (5). By contrast, in classical CHH, profound and sustained deficiency of both LH and FSH leads to absent Sertoli cell proliferation, failure of testicular volume expansion, and—when prolonged—progressive impairment of testicular somatic cell function and germ cell niche establishment. This developmental arrest may underlie the “dual defect” phenotype, characterized by both impaired gonadotropin secretion and intrinsic testicular dysfunction.

A study reported spermatogenesis induction in 64% of 223 adult patients with congenital hypogonadotropic hypogonadism (CHH) following gonadotropin therapy (6). However, treatment failure—manifesting as absent or insufficient spermatogenesis—remains common, and pre-existing or progressive testicular dysfunction, rather than isolated hypothalamic–pituitary insufficiency, may be a key determinant of nonresponse. We hypothesize that prolonged gonadotropin deficiency beyond the mini-puberty and early childhood periods contributes to irreversible functional decline and impaired proliferative capacity of both Sertoli and Leydig cells. Notably, in our clinical cohort, several children diagnosed with suspected dual-defect HH developed signs of testicular insufficiency—including declining inhibin B levels, and/or diminished testosterone response to hCG stimulation—following standard gonadotropin replacement initiated in adolescence. These observations support the concept of a critical therapeutic window during late childhood and early puberty, wherein timely gonadotropin reactivation may preserve or restore intrinsic testicular function. Accordingly, we propose initiating GnRH or gonadotropin therapy during the physiological pubertal timeframe in carefully selected suspected dual-defect HH patients, with close monitoring of endocrine and ultrasonographic parameters; if active spermatogenesis is confirmed, cryopreservation of ejaculated should be pursued without delay. To date, evidence supporting this approach remains limited to anecdotal case reports and small series; cohort studies—and validated, early biomarkers predictive of—are urgently needed.

Anti-Müllerian hormone (AMH) is produced by the immature Sertoli cells and a biomarker for assessing Sertoli cell function during childhood in boy. The hCG stimulation test is a critical diagnostic tool for evaluating testicular function, especially during childhood (7). An hCG prolongation test may further enhance diagnostic accuracy by overcoming the potential blunting of the hypothalamic-pituitary-gonadal (HPG) axis due to hypopituitarism. A post-stimulation serum testosterone level below 100 ng/dL is used as a provisional cutoff to indicate impaired testicular function (8). Previous studies have found that baseline testicular volume (TV) and a history of cryptorchidism serve as significant predictors of spermatogenesis (9–12). Lower serum inhibin B (<60 pg/mL), and variants in ANOS1 are also negative predictor for spermatogenesis (3, 4, 13). However, the predictive value of AMH and the human chorionic gonadotropin (hCG) prolongation stimulation test in the treatment of suspected dual CHH remains unclear.

In this study, we employed the extended hCG stimulation test to characterize intrinsic testicular functional capacity in pediatric CHH patients. We administered early GnRH or gonadotropin therapy to a cohort of suspected dual-defect CHH patients, systematically monitoring treatment response through serial assessments of testicular volume, serum testosterone, inhibin B, and AMH, as well as documented occurrence of nocturnal emissions (adjunct clinical follow-up indicator, not proof of spermatogenesis). Using retrospective analytical methods, we aimed to identify pre-treatment and early on-treatment variables predictive of early treatment response, thereby timely intervention to preserve reproductive potential during the critical developmental window.

## 2 Methods and research subjects

### 2.1 Study design and setting

This retrospective study included patients with suspected dual CHH who received GnRH pump or combined gonadotropin (hCG/hMG) treatment at a single center in Beijing Children’s Hospital from January 1, 2010, to January 1, 2025. We divided the patients into favorable early response and suboptimal early response groups. First, we compared the baseline clinical characteristics of both groups. By constructing receiver operating characteristic curves, we evaluated the predictive efficacy of baseline AMH, INHb, and T levels after hCG prolongation stimulation test, testosterone levels and testicular size during early-stage treatment in predicting the early response of suspected dual CHH patients to GnRH or gonadotropin therapy.

### 2.2 Ethical issues

This study adhered to the Declaration of Helsinki and local regulations. The research protocol was reviewed and approved by the Ethics Committee of Beijing Children’s Hospital. Informed consent forms were provided by all the participants and their guardians.

### 2.3 Research subjects

Inclusion criteria: All suspected dual CHH patients who visited our department and received treatment with GnRH pumps or combined gonadotropins (hCG/hMG) are eligible for inclusion. The diagnostic criteria for CHH and suspected dual CHH were based on articles previously published by our research group (8). Patients who reported hyposmia, those with abnormal olfactory bulb or olfactory tract development indicated by olfactory bulb MRI, and those with a family history of Kallmann Syndrome (KS) were diagnosed with Kallmann Syndrome. Patients with normal olfaction and/or normal olfactory bulb MRI findings were diagnosed with nIHH. Patients with abnormalities in other pituitary hormone axes were diagnosed with multiple pituitary hormone deficiency (MPHD); patients with eye defects, heart malformations, ear malformations, and pathogenic CHD7 gene variants were diagnosed with Charge syndrome.

The exclusion criteria were as follows: patients who did not receive GnRH or gonadotropin therapy, those with a treatment duration of less than 6 months, those treated with testosterone undecanoate, those with incomplete clinical data, and those lost to follow-up.

### 2.4 Hormone detection

Luteinizing hormone (LH), follicle-stimulating hormone (FSH), and testosterone levels were measured using an enzyme-enhanced chemiluminescence immunoassay (Immulite 2000; Siemens Healthcare GmbH, Germany). AMH levels were assessed using an electrochemiluminescence immunoassay. Inhibin B (INHb) concentrations were determined via chemiluminescent immunoassay. The hCG standard, hCG prolonged test, and gene sequencing methods were performed as previously described (11).

### 2.5 Treatment and follow-up of participants

All participants received either a GnRH pump or combined gonadotropin (hCG/hMG) treatment. GnRH pump treatment: 5–12 µg administered every 90 min, delivering 16 pulses over a 24-hour period (1.6–4 µg/kg per pulse). Therapy was initiated at a low dose and subsequently titrated based on serum testosterone levels to maintain concentrations as close as possible to the target range of 200–500 ng/dL. Combined gonadotropin (hCG/hMG) treatment: Method 1: hCG 1000–2000 U was intramuscularly injected every other day or twice a week. Treatment started at a low dose, and the dose was gradually increased until the testosterone level reached 200 ng/dL to start the formal treatment. During the formal treatment phase, 75 U of hMG was added daily. During treatment, the dose of hCG was adjusted to maintain the testosterone value within 200–500 ng/dL as much as possible. Method 2: hCG 500–2000 U was administered 1–3 times a week, and hMG 75–150 U was administered 1–3 times a week; during treatment, the dose of hCG was adjusted to maintain the testosterone value within 200–500 ng/dL as much as possible.

All patients were followed up at the 1st and 3rd months after medication and then followed up every three months for hormone level testing and physical examination. Testosterone levels were measured within 12–24 h of hCG/hMG administration. Serum testosterone concentrations were monitored during continuous GnRH pump use. Testicular size was evaluated using a Prader orchidometer and ultrasound, with the mean value of bilateral testicular volumes used for data analysis.

### 2.6 Assessment of early clinical response to GnRH or gonadotropin therapy

Based on the normal reference range of male testosterone levels and combined with our patient data, the response was defined as follows: Patients who failed to achieve a serum testosterone concentration of ≥200 ng/dL after ≥6 months therapy, had no increase in testicular volume or had no nocturnal emissions during longer follow-up were classified as exhibiting a suboptimal early response. Conversely, those who attained ≥200 ng/dL within 6 months of therapy initiation and/or reported nocturnal emissions during treatment were classified as exhibiting a favorable early response.

### 2.7 Statistical analysis

Statistical analyses were performed using SPSS software version 25.0 and Graphpad Prism 7. Continuous variables with a normal distribution are presented as the mean ± standard deviation (SD), and variables with a non-normal distribution are presented as the median and interquartile range M (QL, QU). Independent-samples t-tests and analysis of variance (ANOVA) were used to compare normally distributed data between groups, while the Mann-Whitney U test was employed for non-normally distributed data. Receiver operating characteristic curves of serum AMH, INHb, and T levels after the hCG prolongation test, testosterone levels during early stages treatment were constructed to determine the optimal cut-off points for judging the early clinical response of suspected dual CHH. The positive likelihood ratio and 95% confidence interval were calculated. Binary Logistic regression analysis was used to analyze the correlations between baseline AMH, cryptorchidism, T levels after the hCG prolongation test and early clinical response of suspected dual CHH. All statistical tests were two-sided, and a P-value < 0.05 was considered statistically significant.

## 3 Results

### 3.1 Participants

**Figure 1:**
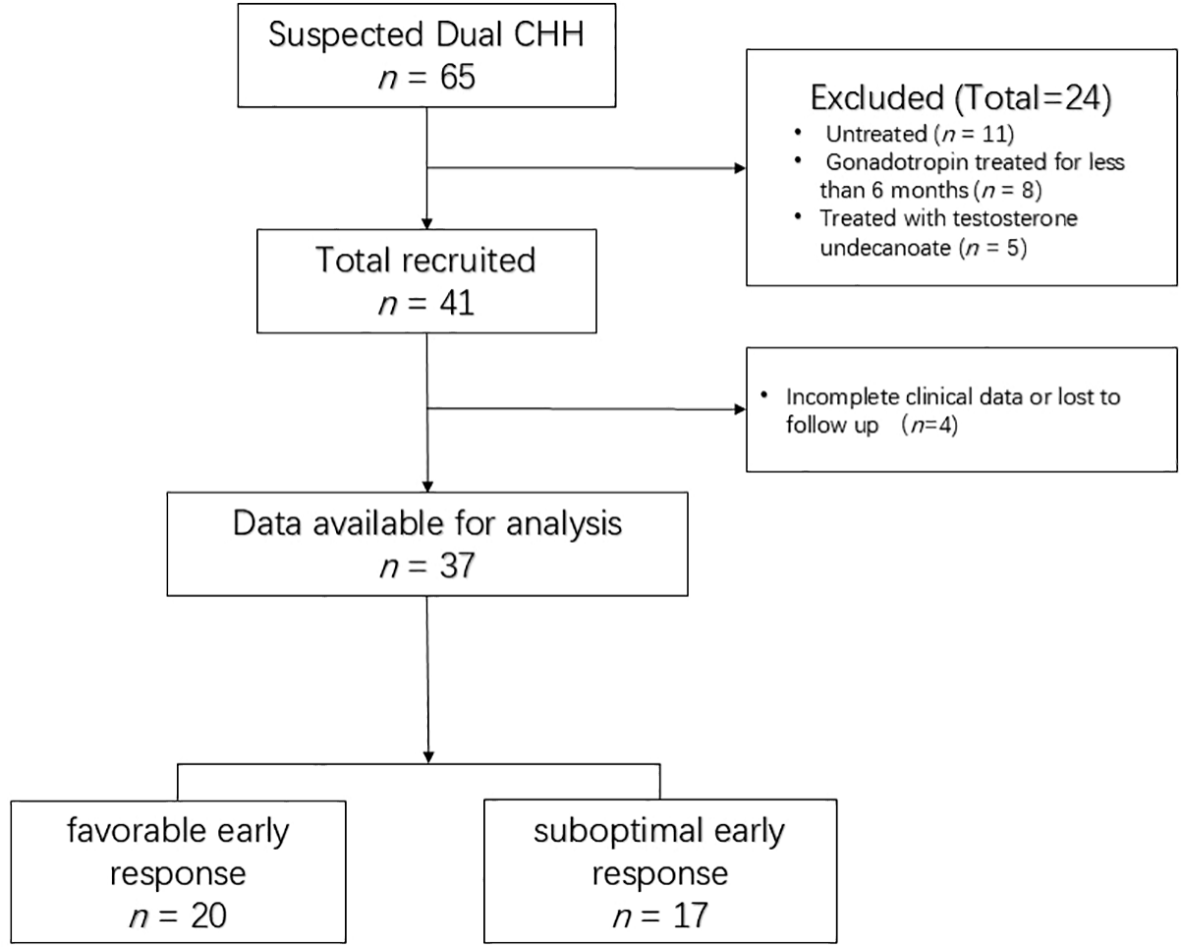
Flow chart of patient enrollment.

A total of 65 patients diagnosed with suspected dual CHH were screened at the research center. A total of 28 patients were excluded, including those who did not receive GnRH or gonadotropin treatments (n = 11), those with a treatment duration of less than 6 months (n = 8), those treated with testosterone undecanoate (n = 5), and those with incomplete clinical data or lost to follow-up (n = 4). Finally, 37 patients met the inclusion criteria and were included in this study (**Figure 1**). Among them, there were 22 cases KS, 2 cases MPHD, 11 cases nIHH, and 2 cases Charge syndrome. The average age of the patients was 13.75 ± 1.96 years. A total of 26 patients underwent genetic testing, and 16 of them carried CHH-related gene mutations. Among them, 12 patients received GnRH pump treatment, 14 received combined method 1, and 11 received combined method 2. The average follow-up time of all patients was 14.75 ± 8.28 months. There was no statistically significant difference in the incidence of favorable early response among the three treatment modalities (**Supplementary Table 1**).

### 3.2 Comparison of baseline clinical data among patients with different treatment responses

Compared with the favorable early response group, the suboptimal early response group had a higher incidence of cryptorchidism, smaller baseline testicular volume measured by ultrasound, lower baseline AMH level, and lower T level after the hCG prolongation stimulation test. In addition, the level of inhibin B was also lower in the suboptimal early response group. Thirteen people in the favorable early response reported nocturnal emissions (Only five patients provided semen test reports confirming sperm production, and two patients underwent sperm cryopreservation.), while none in the suboptimal early response group did. There were no significant differences between the two groups in terms of age, bone age, height, weight, basal LH, FSH, or testosterone levels after the standard hCG stimulation test (shown as **Table 1**).

**Table 1:** Baseline Features of suspected dual CHH patients with different responses.

Items	Favorable early response (n=20)	Suboptimal early response (n=17)	P
Age (y)	13.71 ± 1.46	13.46 ± 2.70	0.741
Bone age (y)	12.89 ± 1.33	12.56 ± 2.11	0.656
Height (cm)	160.15 ± 7.34	155.05 ± 9.15	0.146
Weight (kg)	56.5 ± 13.03	50.98 ± 9.60	0.218
Cryptorchidism(n)	8	13	0.065
Micropenis (n)	13	13	0.800
KS (n)	10	12	0.385
nIHH (n)	7	4	0.331
MPHD (n)	1	1	0.969
Charge syndrome (n)	1	1	0.969
Penile length (cm)	4.84 ± 1.64	3.83 ± 0.89	0.063
TV measured by Prader orchidometer (ml)	1.96 ± 1.12	1.35 ± 0.72	0.066
TV measured by ultrasound (ml)	0.43 ± 0.34	0.18 ± 0.09	0.046
Baseline LH (IU/L)	0.10 (0.01, 0.15)	0.10 (0.01, 0.13)	0.635
Baseline FSH (IU/L)	0.54 (0.35, 0.84)	0.55 (0.41, 0.78)	0.505
Baseline LH/FSH	0.13 (0.02, 0.37)	0.14 (0.00, 0.27)	0.371
Baseline T (ng/dl)	16.48 ± 7.23	17.55 ± 10.67	0.741
Baseline AMH (ng/mL)	15.39 ± 5.60	6.84 ± 4.62	0.000
Baseline INHB (pg/mL)	34.8 ± 26.66	20.83 ± 8.32	0.065
T after hCG test (ng/dl)	32.87 ± 23.40	26.96 ± 11.12	0.408
T after prolonged hCG test (ng/dl)	70.52 ± 20.21	28.80 ± 19.30	0.000
nocturnal emissions (n)	13	0	0.000

KS, Kallmann Syndrome; nIHH, normosmic isolated hypogonadotropic hypogonadism; MPHD, multiple pituitary hormone deficiency; TV, Testicular volume; LH, luteinizing hormone; FSH, follicle-stimulating hormone; T, testosterone. AMH, Anti-Müllerian hormone; hCG, human chorionic gonadotropin; INHB, Inhibin B.

### 3.3 Logistic regression analysis to evaluate the related factors of treatment response

To preliminarily explore the factors associated with the early treatment response to GnRH or gonadotropin treatment in suspected CHH patients, we conducted a binary Logistic regression analysis with the presence of a favorable early response as the dependent variable and cryptorchidism, baseline AMH, testicular volume, and T level after the hCG prolonged stimulation test as independent variables. The results indicated that the level of baseline AMH was positively correlated with a favorable early response (odds ratio = 1.977; 95% confidence interval: 1.010–3.870; p = 0.047).

### 3.4 Construct ROC curve to evaluate the performance of testis-related hormones in predicting the response of suspected dual CHH patients to fertility induction treatment

**Figure 2:**
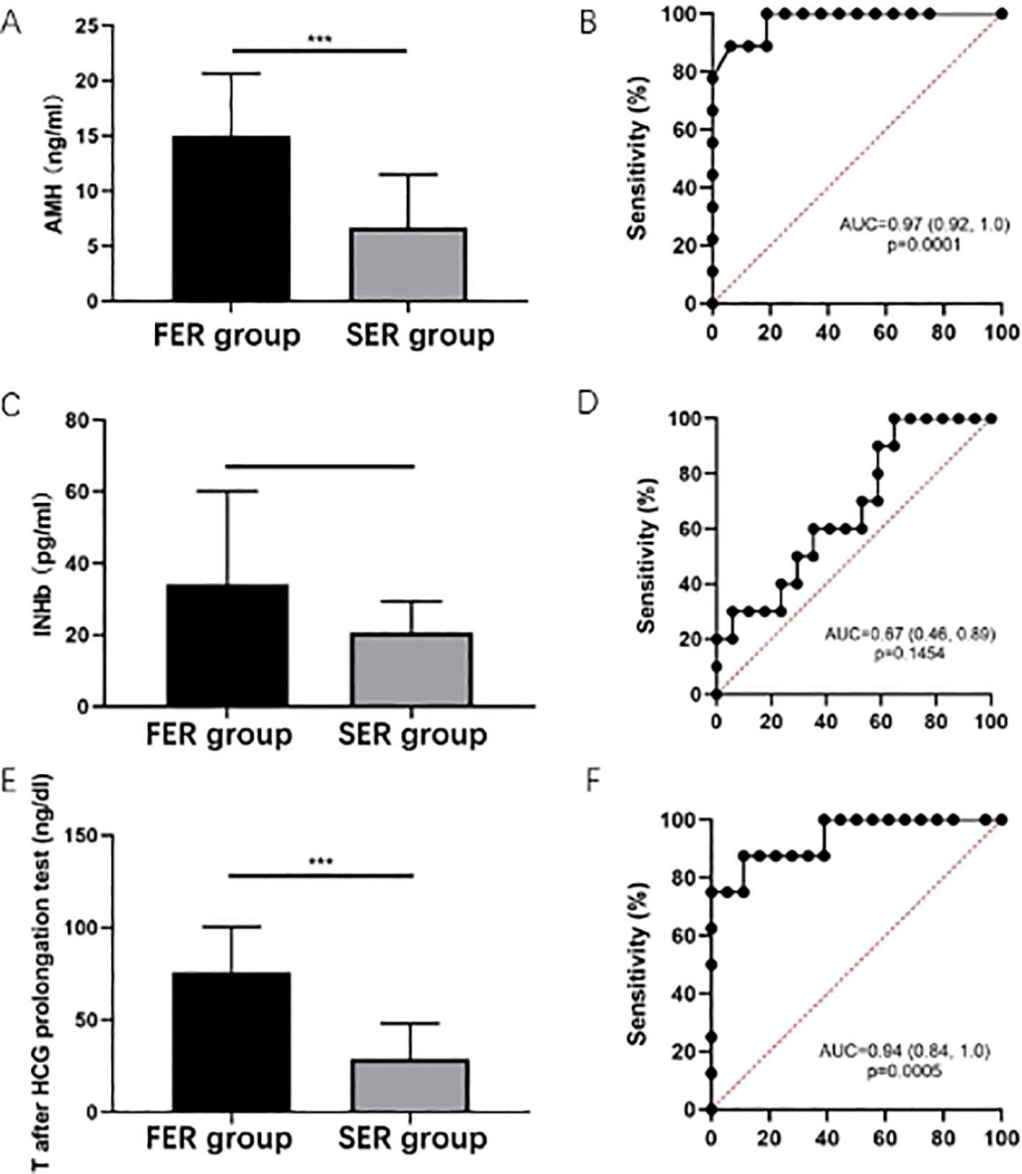
ROC curve analysis for patients with suspected dual CHH based on baseline clinical characteristics: **(A)** AMH levels of two groups; **(B)** ROC Curve analysis of baseline AMH; **(C)** INHb levels of two groups; **(D)** ROC curve analysis of baseline INHb; **(E)** T after hCG prolongation test of two groups; **(F)** ROC curve analysis of T after hCG prolongation test; FERgroup, favorable early response; SERgroup, suboptimal early response groups. AUC, area under curve ***, p< 0.001.

To identify indicators that can preliminarily distinguish early treatment responses, we further constructed an ROC curve to evaluate the value of AMH, inhibin B, and T levels after the hCG prolongation stimulation test in predicting the therapeutic response. The results are summarized in **Table 2**; **Figure 2**. The AUC of AMH and testosterone after the hCG prolongation stimulation test was higher, with better sensitivity, specificity, and positive likelihood ratio. When the cutoff value of AMH was 8.9 ng/ml, the sensitivity and specificity were 88.89% and 93.75%, respectively. When the cut-off value of testosterone after the hCG prolongation stimulation test was 51.95 ng/dl, the sensitivity and specificity were 87.5% and 88.89%, respectively.

**Table 2:** ROC Curve analysis for patients with suspected dual CHH based on baseline clinical characteristics.

Items	Cutoff	Sensitivity (%, 95% CI)	Specificity (%, 95% CI)	+LR
AMH (ng/mL)	8.9	88.89 (56.5, 99.43)	93.75 (71.67, 99.68)	14.22
INHB (pg/ml)	30.94	90 (59.98, 99.49)	41.18 (21.61, 63.99)	1.53
T after hCG prolongation test (ng/dl)	51.95	87.5 (52.91, 99.36)	88.89 (67.20, 98.03)	7.875

AMH, Anti-Müllerian hormone; hCG, human chorionic gonadotropin; INHB, Inhibin B.

### 3.5 ROC curve analysis based on clinical data collected during the treatment process

**Figure 3:**
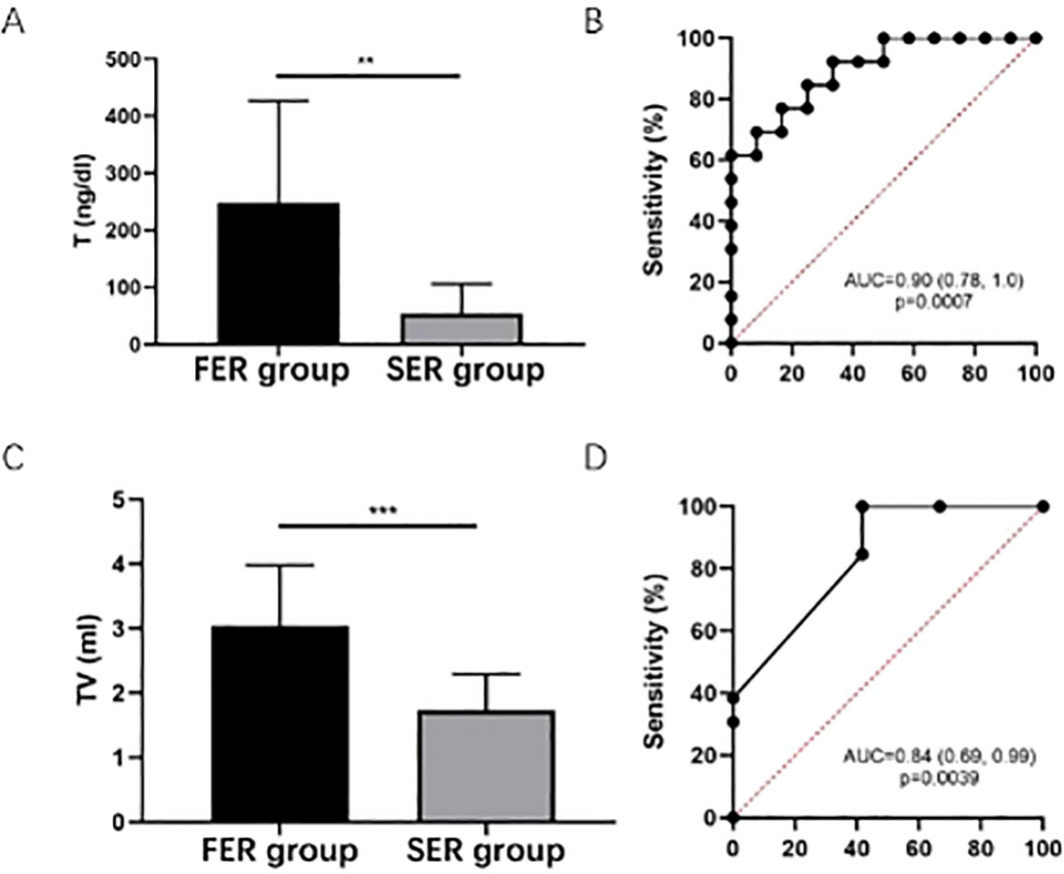
ROC curve analysis based on clinical data collected during the treatment process: **(A)** T levels of two groups; **(B)** ROC Curve analysis of T; **(C)** TV of two groups; **(D)** ROC Curve analysis of TV. FERgroup, favorable early response; SERgroup, suboptimal early response groups. AUC, area under curve. T, testosterone; TV, testicular volume ***, p< 0.001.

To evaluate the efficacy of clinical parameters in preliminarily distinguish early treatment responses, we selected follow-up data from all patients at time points when hCG or GnRH dosages were as comparable as possible based on the cumulative doses administered during the 1- to 6-month follow-up period and calculated the range of these cumulative dosages. Therefore, we performed ROC curve analysis based on the testosterone levels and testicular volume measurements of patients when the cumulative dosages of hCG, hMG, and GnRH were 24,000–40,000 U, 0–3,375 U, and 4.0–4.8 mg, respectively. The AUC of the testosterone value for prediction was larger than that of testicular volume. When the testosterone level was 135.8 ng/dl, the sensitivity for predicting a favorable early response was 92.31%, and the specificity was 66.67%. The details was shown in **Table 3** and **Figure 3**.

**Table 3:** ROC curve analysis based on clinical data collected during the treatment process.

Items	Cutoff	Sensitivity (%, 95% CI)	Specificity (%, 95% CI)	+LR
T (ng/dL)	135.8	92.31 (66.69-99.61)	66.67 (39.06, 86.19)	2.77
TV (ml)	3.0	100 (77.19, 100)	58.33 (31.95, 80.67)	2.40

TV, Testicular volume; T, testosterone.

### 3.6 Gene variants related to HH in patients with different treatment responses

**Figure 4:**
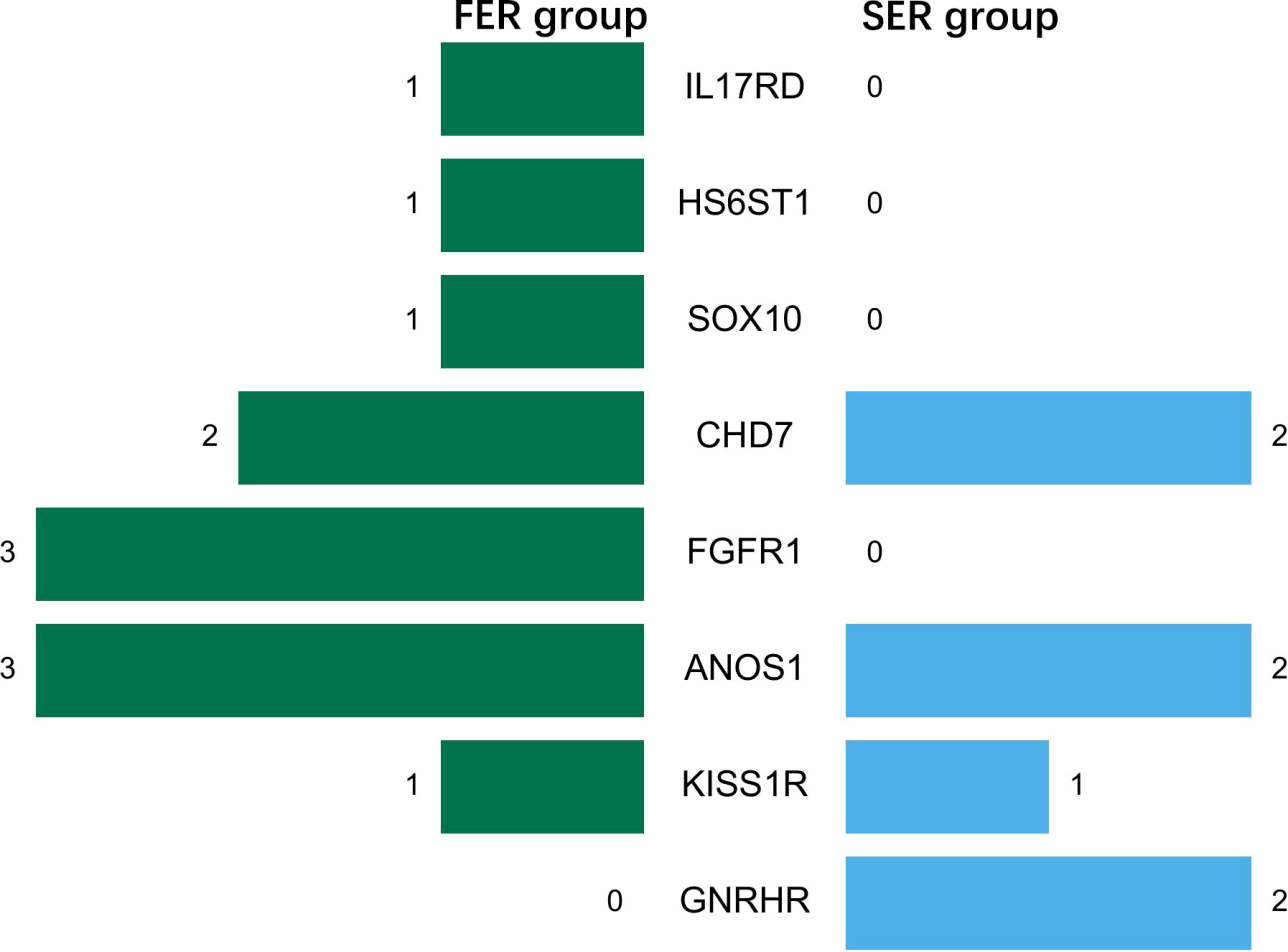
Gene variants related to HH in patients with different treatment responses. FERgroup, favorable early response; SERgroup, suboptimal early response group.

A total of 26 children underwent whole-exome gene testing. Among them, 10 cases (10/15) in the favorable early response group were detected with gene variants related to HH (possibly pathogenic or uncertain), and 6 cases (6/11) in the suboptimal early response group were detected with gene variants related to HH (possibly pathogenic or uncertain). No significant difference was observed in the positivity rates between the two groups (p = 0.531). The frequency of FGFR1 gene variants was slightly higher in the favorable early response group (p = 0.263). Two oligogenic mutations were identified in the favorable early response group: IL17RD p.Asn503Ser, SOX10 p.Met596Thr, and CHD7 p. Lys2331Glu, ANOS1 p.Asp286Asn. In contrast, only one oligogenic mutation was observed in the suboptimal early response group: ANOS1 p.Trp231Ter and CHD7 p.Asp1638Glu mutations (shown as **Figure 4**).

## 4 Discussion

In this study, we presented the early clinical response and potential predictive factors in 37 pediatric patients with suspected dual CHH who underwent GnRH or gonadotropin treatment. Our findings suggested that baseline AMH was positively correlated with a favorable early response. In addition, baseline AMH and T levels after the hCG prolongation stimulation test maybe can used as indicators to preliminarily distinguish early treatment responses. Since semen analysis was performed only in partial patients in this study, we cannot make a final confirmation of dual defect. Our results only presented the early clinical response of suspected dual CHH.

We conducted puberty induction therapy in 37 male patients with suspected dual CHH. Of these, 20 patients achieved normal mid-range T concentrations within six months of treatment initiation, and 13 reported nocturnal emissions, in whom five patients provided semen test reports confirming sperm production, and two patients underwent sperm cryopreservation. These individuals exhibited relatively greater baseline testicular volumes, higher baseline AMH levels and T levels after hCG prolongation stimulation test. These findings suggested that despite the presence of suspected dual defects, partial testicular function is preserved, warranting consideration of spermatogenic induction therapy. Sykiotis et al. similarly reported that some patients with dual defects achieved spermatogenesis and attained normal testosterone levels following GnRH therapy (3). An Italian team published a retrospective study that included four CHH patients with severe GnRH deficiency (TV, 2–3 mL) who received sequential therapy. Therapy significantly increased TV, and three of four patients achieved sperm in their ejaculate (14). A recent comprehensive review recommended GnRH pulse therapy or combination therapy for male patients with CHH and severe GnRH deficiency; however, the optimal therapeutic strategy remains to be established (15). Case reports studies showed rFSH pretreatment may improve sperm production in HH patients with small testicular volume. In this study, puberty was induced with either hCG followed by hMG or combined hCG/hMG. However, this study is a single-center retrospective study with a long inclusion period, and the treatment plans were mainly based on clinical practice and individual conditions at that time. A uniform rFSH priming strategy was not implemented for all patients with small testicles, which indeed constitutes an important limitation of the study design. And for patients with small baseline TV, future prospective studies should incorporate a standardized FSH priming strategy to more accurately assess its impact on treatment response.

AMH, produced by immature Sertoli cells, serves as a key biomarker for evaluating early testicular function in infants and young boys and complements inhibin B as a marker of Sertoli cell activity (16). AMH levels peak at approximately one year of age and remain relatively high throughout childhood (17). During this period, AMH expression reflects the baseline functional status of Sertoli cells and facilitates reactivation of the HPG axis at puberty (18). Our study demonstrated that baseline AMH levels were the most significant predictor of therapeutic response in patients with suspected dual CHH, indicating that Sertoli cell function plays a critical role in spermatogenesis.

However, we also found that T levels measured after the hCG prolongation stimulation test had significantly difference between two group. Under normal physiological conditions, elevated LH levels stimulate Leydig cell differentiation and testosterone production. Increased intratesticular testosterone, together with elevated FSH levels, initiates and sustains spermatogenesis (15, 19, 20). Intratesticular paracrine testosterone concentrations are essential for this process and can be 30–100 times higher than systemic endocrine levels (21). A study conducted on men with hypogonadotropic hypogonadism demonstrated that the combination of FSH and hCG-induced T effectively stimulated sperm production, whereas the administration of FSH alone or exogenous testosterone failed to achieve the same outcome (22). High level of T levels after the hCG prolongation stimulation test in favorable early response group highlight the importance of both Sertoli and Leydig cell functions in spermatogenesis in children with suspected dual CHH.

In addition, our findings provided a reference for the selection of combined therapy for puberty induction in patients with CHH. In individuals with a small TV and baseline AMH level below 8.9 ng/mL, rFSH pretreatment may be prioritized to promote Sertoli cell proliferation and maturation, followed by the addition of low-dose hCG with an extended treatment duration to preserve testicular function. Conversely, in patients exhibiting bigger TV, higher baseline AMH levels and a testosterone hCG prolongation stimulation test, early initiation of combined hCG and hMG therapy may be considered, potentially shortening the treatment course, improving patient adherence, and ultimately enhancing therapeutic outcomes. These results offer a basis for individualized treatment strategies for CHH.

Puberty induction therapy can be attempted in all patients with suspected dual CHH. By observing the changes in indicators in the early stages of treatment and predicting the treatment effect, more precise treatment options can be provided for patients. We analyzed T levels and mean TV at certain doses of hCG/hMG and GnRH following treatment initiation and found that T concentrations exceeding 135.8 ng/dL and a TV greater than 3 mL were associated with favorable early response. The length of the seminiferous tubules is determined by Sertoli cell mass (23, 24), and these tubules constitute approximately 80- 90% of the testicular volume (25). Therefore, it serves as an indicator of Sertoli cell quantity. Sertoli cell proliferation is critical for reproductive potential (15, 26), as each mature Sertoli cell can support a limited, species-specific number of germ cells (21). Therefore, an increase in testicular volume during treatment indicates the preservation of spermatogenic capacity in the testis. Hence, T levels and mean TV during the initial stage of treatment may serve as useful indicators for predicting therapeutic outcomes during treatment.

Finally, there was no significant difference in the frequencies of genes associated with HH between the two response groups. Two previous studies found that patients with IHH carrying FGFR1 gene variants had a high sperm occurrence rate after spermatogenesis-promoting treatment (27, 28). Our study found that FGFR1 gene mutations occurred only in the favorable early response group. Our findings once again demonstrate that variations in the FGFR1 gene are associated with a favorable prognosis.

This study had several limitations. Firstly, as a retrospective analysis, it is inherently susceptible to bias and the absence of upfront rFSH priming in the treatment protocol may have influenced therapeutic outcomes and contributed to the poor results documented in the suboptimal early response group. Secondly, a subset of patients remained under ongoing treatment, and the assessment of therapeutic efficacy was primarily based on early clinical response, such as changes in testosterone levels during treatment, without a comprehensive evaluation of the final sperm production outcomes. Thirdly, considerable inter-individual variability in medication dosing among patients precluded meaningful comparisons of pubertal induction responses under standardized dosage regimens. Furthermore, owing to the rarity of the condition, the study was limited by a relatively small sample size. Therefore, ROC curve analysis and Logistic analysis had the risk of overfitting and instability. Thus, the results of this study were only preliminary explorations. Further analysis with an increased sample size and balanced baseline characteristics is needed to confirm the research conclusions. Fourthly, the patients included in this study received three different treatment protocols, which might have affected the results and the interpretation of the predictive efficacy of the biomarkers due to the heterogeneity of the treatment protocols. We conducted a preliminary exploratory analysis of the treatment responses among the three treatment groups and found no significantly difference of treatment responses in those three groups (see **Supplementary Table 1**). Due to the small sample size in each treatment group, and the incomplete balance of baseline characteristics and cumulative doses, we conducted additional exploratory comparisons, but these should not be regarded as definitive conclusions. Finally, as the study was conducted in a pediatric hospital, facilities for semen analysis were unavailable; therefore, semen testing was only performed in partial patients reporting nocturnal emissions. Hence, this article cannot make a final confirmation of dual defect and should not be interpreted as a direct evaluation of spermatogenesis.

## 5 Conclusion

GnRH or gonadotropin treatment may facilitate testicular development in suspected dual HH patients. In this exploratory pediatric cohort with suspected dual CHH, baseline AMH and testosterone after the hCG prolongation test may associated with early response to GnRH and gonadotropin therapy. These findings may help stratify treatment responsiveness, but require validation in larger cohorts with standardized treatment protocols and objective semen-based endpoints.

## Data Availability

The original contributions presented in the study are included in the article/**Supplementary Material**. Further inquiries can be directed to the corresponding author.

## Supplementary material

Supplementary Table 1 Clinical features of suspected dual CHH patients with different treatments.
