# Supplementary material for: Predictors of early response to GnRH and gonadotropin therapy in pediatric patients with suspected dual congenital hypogonadotropic hypogonadism: a retrospective single-center study
Source: Front Endocrinol (Lausanne). 2026 May 29;17:1844974. doi: 10.3389/fendo.2026.1844974 (PMC13259665; doi:10.3389/fendo.2026.1844974)
Supplement: Supplementary Table 1 — Clinical features of suspected dual CHH patients with different treatments. [file Table1.docx]

Table S1 Clinical Features of suspected dual CHH patients with different treatments

| Items | **GnRH pump (n= 12)** | **Combined method 1（n= 14）** | **Combined method 2 (n=11)** | P |
| --- | --- | --- | --- | --- |
| Age (y) | 13.72 ± 1.70 | 13.88 ± 1.71 | 13.66 ± 2.41 | 0.958 |
| Bone age (y) | 13.28 ± 1.07 | 12.50 ±1.56 | 12.94±2.01 | 0.640 |
| Cryptorchidism(n) | 5 | 7 | 9 | 0.883 |
| TV measured by Prader orchidometer (ml) | 1.70 ± 0.94 | 1.97±1.26 | 1.39±0.71 | 0.344 |
| Baseline AMH (ng/mL) | 12.94 ± 7.19 | 15.43±6.89 | 10.03±6.23 | 0.185 |
| Baseline INHB (pg/mL) | 21.05 ± 5.18 | 28.09±15.92 | 33.31± 29.67 | 0.544 |
| T after prolonged hCG test (ng/dl) | 44.99 ± 32.89 | 60.24 ± 22.41 | 62.29±27.28 | 0.483 |
| Favorable early response (n) | 5 | 9 | 6 | 0.137 |
